# Feeding cancer to death - a triad of aromatic acids reduces tumor growth

**DOI:** 10.1038/s41418-024-01372-9

**Published:** 2024-09-12

**Authors:** Audrey Xavier, Ivan Dikic

**Affiliations:** 1 Institute of Biochemistry II, Faculty of Medicine, Goethe University Frankfurt, Frankfurt am Main, Germany; 2 Buchmann Institute for Molecular Life Sciences, Goethe University Frankfurt, Frankfurt am Main, Germany; 3 Max Planck Institute of Biophysics, Frankfurt am Main, Germany

**Keywords:** Proteasome, Ubiquitylation

**Fig. 1:**
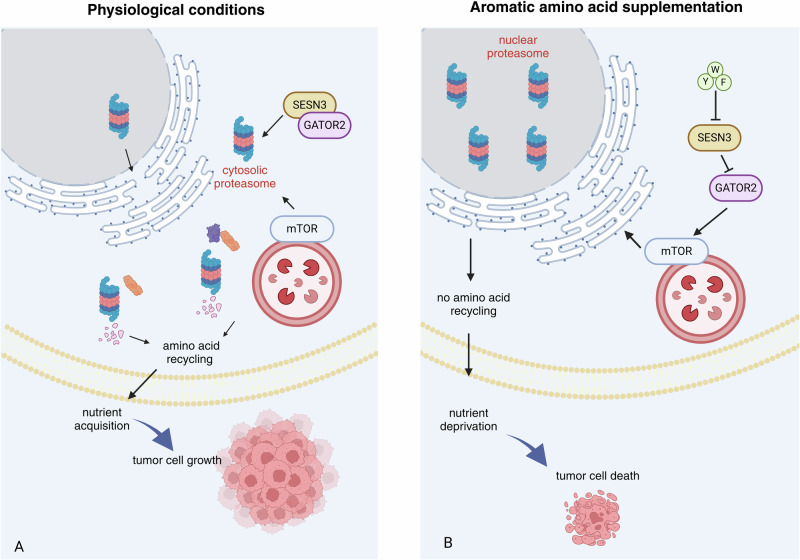
Aromatic amino acid supplementation induces tumor cell death. **A** During physiological conditions Sestrin3 interacts with and inhibits GATOR2 and mTOR mediated proteasome shuttling. This allows the proteasome to transcolate to the cytosol, replenish nutrients by proteolysis and facilitate tumor growth, **B** Supplementation of the aromatic amino acids Tyr, Trp, and Phe (YWF) to tumor cells activates mTOR, a signal mediated via a novel sensor, Sestrin3. This activation results in sequestration of the proteasome in the nucleus, inhibition of cytosolic proteolysis, and subsequent tumor cell death.

Cancer cell proliferation is coupled with altered cellular signaling and metabolic adaptation to support tumor progression. Changes in metabolism can be specific to cancer growth and directly transform a healthy cell into tumor precursors. However, not all metabolic alterations necessarily contribute equally to cellular transformation to tumor cells [1]. To this date, many cancer therapies rely on unspecific inhibitors of cellular proliferation. Thus, identifying key regulators of tumor-specific metabolic signaling pathways can aid in improving anticancer therapies. In this issue, Livneh et al., present a novel strategy to induce selective apoptosis of tumor cells by exploiting a specific metabolic regulation of proteasome sequestration to the nucleus [2] (Fig. 1A, B).

A key player regulating metabolic processes and the cellular response to nutrient stress is the serine/threonine kinase mechanistic target of rapamycin (mTOR). Extensive research has uncovered mTOR as a major facilitator of amino acid, glucose, lipid and fatty acid as well as nucleotide metabolism [3]. Commonly, mTOR is activated by increased serum levels of amino acids (such as glutamine, arginine and leucine) simply due to degradation of dietary proteins. Amino acid stimulation converts Rag GTPases to their active state which tether mTOR to the lysosomal membrane stabilizing its activation [4]. The essential complexes GATOR1 and GATOR2 receive signals from cytosolic leucine and arginine and regulate mTOR activity [5]. GATOR1 inhibits mTORC1 signaling by acting as a nucleotide exchange factor for RagA/B. GATOR2 is a positive regulator of mTORC1 signaling that interacts with GATOR1 at the lysosomal membrane [5, 6]. Researchers achieved a breakthrough by the discovery of Sestrin2 as a direct leucine sensor and GATOR2 interacting protein that inhibits mTORC1 signaling in the absence of leucine [7, 8]. Previously, Livneh et al. newly identified Sestrin3 as a sensor of the aromatic amino acids tyrosine, phenylalanine, and tryptophan (YWF). Administering YWF activates mTOR via Sestrin3, which in turn inhibits GATOR2 [9]. While several mTOR targeting drugs were developed to treat cancer progression many challenges remain, including insufficient phosphorylation blockage of mTOR targets and compensatory upregulation of alternative proliferative pathways. Thus, leading to a cytostatic not cytotoxic effect. However, Livneh et al. demonstrate that mTOR activation in the context of YWF leads to nuclear sequestration of the proteasome ultimately yielding in cell death of highly proliferative cancer cells (Fig. 1A, B).

The cell governs protein degradation via the 26S proteasome, which is central to the ubiquitin-proteasome system (UPS). Researchers have spent over three decades to unravel its composition, function and regulation [10, 11]. As an essential part of the cellular quality control machinery the proteasome remains a major target for drug discovery. Based on recent advances through structural studies many research avenues opened into developing proteasome-targeting compounds such as inhibitors for treating multiple myeloma [12] and other cancer types or activators that could change the outcome of neurodegeneration and aging [13]. While the proteasome has essential functions in maintaining protein homeostasis in the cytosol, an equally important portion can be found in the nucleus [14, 15]. Upon amino acid shortage, the proteasome is known to replenish the nutrient shortage by degradation of preexisting proteins. Inhibition of the proteasome during amino acid nutrient deprivation markedly impaired translation [16]. As noted, in a previous study, Livneh et al. showed that the proteasome can be kept in the nucleus by feeding the cells with YWF. Now, they exploit this mechanism of shuttling between nucleus and cytosol in vivo by confining proteasomes in the nuclei, inhibiting amino acid recycling and thus specifically forcing the highly metabolic cancer cells into death.

However, proteasome shuttling to the cytosol is not limited to amino acid starvation but also occurs during hypoxia, a defining characteristic of cancer cells. Hypoxia-inducible factors are already an important therapeutic target in cancer research [17], whereas feeding the aromatic amino acids YWF to tumor cells directly is a unique and novel approach. Ciechanover and colleagues show that administering YWF not only upregulates transcripts involved in apoptosis, but also downregulates metabolism of macromolecules, proteins, carbohydrates and pro-tumorigenic pathways. Excitingly, they further explore this mechanism in vivo by treating YWF to human breast and uterine cervix tumor xenograft models in mice, which show proteasome sequestration in the nucleus in vivo as well as a significant reduction in tumor tissue already 4 days post treatment. Here, the type of administration does not play much of a role, as a direct injection of YWF to the tumor’s bed was just as effective as dissolving it in drinking water. This is a promising perspective for non-invasive cancer treatment. As xenografts have limitations in mimicking actual endogenous tumors the authors tested their hypothesis on endogenous epithelial tumor models such as colorectal cancer and bladder carcinoma, which both show significantly reduced tumor mass upon YWF treatment. Even for mesenchymal tumors such as carcinogen-induced sarcoma model, YWF treatment resulted in reduced tumor size and proteasome sequestration in the nucleus. Most astonishingly however, YWF treatment not only showed reduced tumor growth but also reduced metastases in the aggressive and rapidly metastasizing 4T1 triple-negative breast cancer model. In many treated cancer patients returning metastases lead to rapid lethality, thus the ability to treat metastasizing cancer is invaluable.

Cieachanover and colleagues’ work paves a new avenue in non-inverse cancer therapy with broad applicability to different cancer types. While YWF treatment alone can reduce tumor growth, since the triad targets a novel, ‘non-canonical’ pathway, the combination with other cancer type specific drugs may turn out to be beneficial in fighting malignancies. While other general proteasome inhibitors are already used in treatment, a potential challenge may be the high toxicity of attenuating an essential cellular machinery. Thus, simply confining the proteasome in a cellular sub compartment may provide a greater dosage range for a variety of malignancies. The authors also introduce SESN3 as a sensor of cellular YWF levels and potential oncogene, as knock-down of SESN3 leads to reduced tumor growth as well. This provides the scientific community yet with another potential therapeutic target. However, how SESN3 senses YWF and if it contributes to oncogenesis has remained unclear. Furthermore, the exact mechanism how proteasome sequestration in the nucleus occurs and why replenishing other mTOR activating amino acids shows no effect regarding proteasome localization requires further investigation. For now, this study provides a novel promising weapon in the armory of anti-cancer therapies.
